# A Description of Skeletal Manifestation in Adult Case of Morquio Syndrome: Radiographic and MRI Appearance

**DOI:** 10.1155/2012/324596

**Published:** 2012-07-05

**Authors:** Annalisa Di Cesare, Alessandra Di Cagno, Stefano Moffa, Paolucci Teresa, Innocenzi Luca, Arrigo Giombini

**Affiliations:** ^1^Operative Complex Unit of Physical Medicine and Rehabilitation, Policlinico Umberto I Hospital, Piazzale Aldo Moro 5, 00185 Rome, Italy; ^2^Department of Medicine and Healt Sciences, University of Molise, Via Francesco De Sanctis, 86100 Campobasso, Italy; ^3^Department of Imaging BIOS Spa, Via Domenico Chelini 39, 00100 Rome, Italy

## Abstract

We report on a rare case of Morquio syndrome, an autosomal recessive mucopolysaccharidosis including type IVA, a deficiency of N-acetylgalctosamine-6-sulfatase and type IVB a deficiency of **β**-galactosidase. A 43-year-old female patient affected by IVB Morquio syndrome underwent instrumental investigation. Conventional plain films of the entire spine, pelvis, chest and knees together with magnetic resonance imaging of the entire column, hip, knees, and ankles demonstrated the characteristics of skeletal changes of this disease. The main abnormalities were platyspondily and hypoplasia of the odontoid process, genua valga deformity and severe multiple degenerative changes of the hips, knees, and ankle joints. Radiographs and above all magnetic resonance imaging are crucial to provide substantial information about the gravity, evolution of the skeletal and joints changes, and the rehabilitation strategies to be followed.

## 1. Introduction

Morquio syndrome is a rare inherited disorder of mucopolysaccharide catabolism. It is an autosomal recessive mucopolysaccharidosis (MPS), which includes the type IV A, a deficiency of N-acetylgalactosamine-6-sulfatase, and the type IV B, a deficiency of beta galactosidase, each resulting in a defective degradation of keratan sulfate [1].

In 1929, Morquio a pediatrician in Uruguay and Brailsford a radiologist in England independently and simultaneously described what is known as Morquio-Brailsford syndrome [2]. In the early 1930s, Husler as stated by Kuy, coined the term dysostosis multiplex to describe the constellation of skeletal findings specific to patients with MPS and other lysosomal storage disorders [3].

The Morquio syndrome in its variants, is characterized by severe skeletal changes, which include hypoplasia of the odontoid process, short neck, barrel chest with pectus carinatum, thoracic kyphoscoliosis, and dwarfism [4]. Other characteristics are joint laxity, dental abnormalities, acoustic deafness, together with cardiac abnormalities, and respiratory problems. Some of X-rays features of Morquio's disease include wide flaring of the ilium, shallow acetabula, flattening of femoral heads, coxa and genua valga, and dysostosis multiplex, while skeletal abnormalities of the spine are platyspondyly with central beaking, hypoplasia, or absence of the odontoid process and kyphosis [5]. Historically, type IV A was considered to have more severe manifestations than type IV B. Life expectancy for patients with the type IV A is normally less than 30 years, but isolated cases of long survival have been documented [6]. These patients survive into adult life, possibly because they developed ossification of the odontoid peg and C1 ring to a variable extent [7].

The diagnosis is usually made on the physical and radiological features, blood enzymes, and a skin biopsy. Excessive urinary levels of keratin sulfate are confirmatory. Except for a recent study of Kitzing and Allman, who reported scintigraphic features in a case a Morquio's syndrome, no other studies have been recently published presenting a systematic imaging collection of this rare disease [8]. The aim of our study is therefore to describe the unique radiographic and MRI associated skeletal deformities in an adult case of type IV B Morquio syndrome. 

## 2. Case Presentation

The clinical history of an Italian female patient of 43 years old with a height of 1,50 cm, begins at two years when after a normal postnatal development, was admitted the first time to a children's hospital because of a delay in growth and difficulty walking but with no mental retardation. At the age of 7 years, she had the second admittance to the hospital for a diagnostic setup, because the growth retardation was apparent, and she referred joints stiffness; physical examination showed muscular weakness, pectus carinatum, stubby neck, genu valgum, joints out of proportion to her age, and globose abdomen. The urine test was positive for the presence of keratan sulfate and fibroblasts cultures obtained from skin biopsies showed a deficiency of beta galactosidase activity confirming the diagnosis of type IVB Morquio syndrome. At the age of 11 years, she underwent tibial varus osteotomy of the left knee, while at 29 years she underwent arthroscopy of the left knee for a medial meniscus regularization procedures, cartilage debridement, and removal of an osteochondral fragment. At 35 years, she underwent surgery for uterine fibromyoma. The patient did not follow any supportive measures or rehabilitation protocol to treat the skeletal manifestations of her disease. 

**Figure 1:**
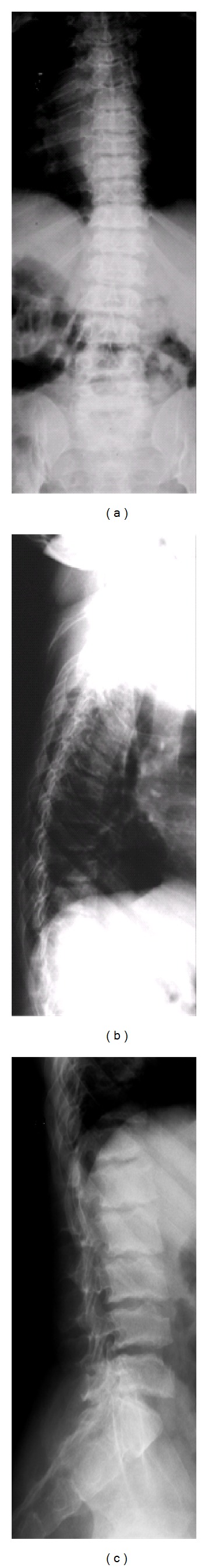
(a) Frontal RX view of the chest depicting bell-shaped chest and decreased height of the trunk. (b) XR lateral view of the thoracic spine depicting varying degrees of platyspondyly with anterior wedging of vertebral bodies. (c) XR lateral view of the lumbar spine with irregularity of the end plates.

**Figure 2:**
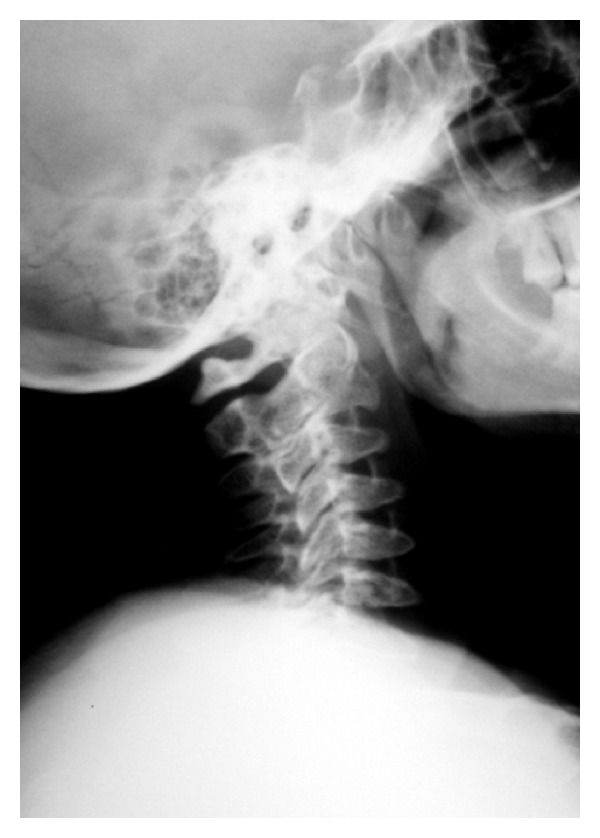
XR lateral view of the cervical spine with wedge shaped vertebral bodies and hypoplasia of the odontoid process.

**Figure 3:**
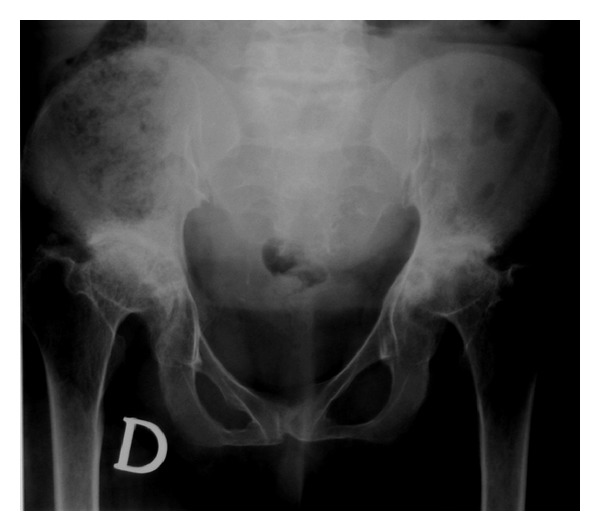
Standing anteroposterior XR view of the pelvis showing short femoral necks and severe degenerative changes of the hip joints.

**Figure 4:**
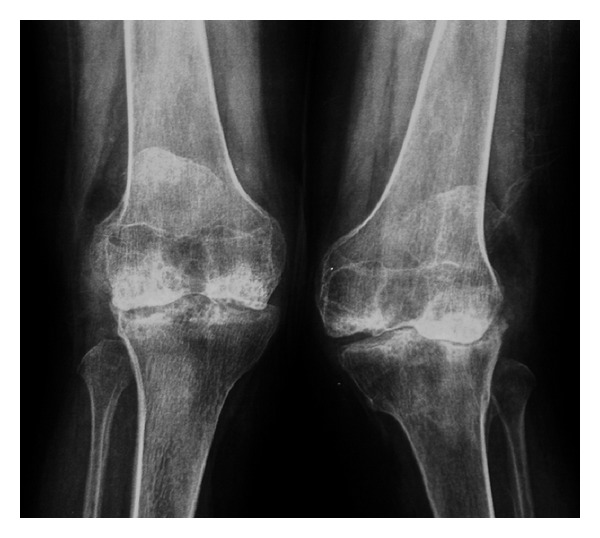
Standing anteroposterior XR of the knees showing genua valga with overgrowth of both medial femoral condyles and severe degenerative changes.

**Figure 5:**
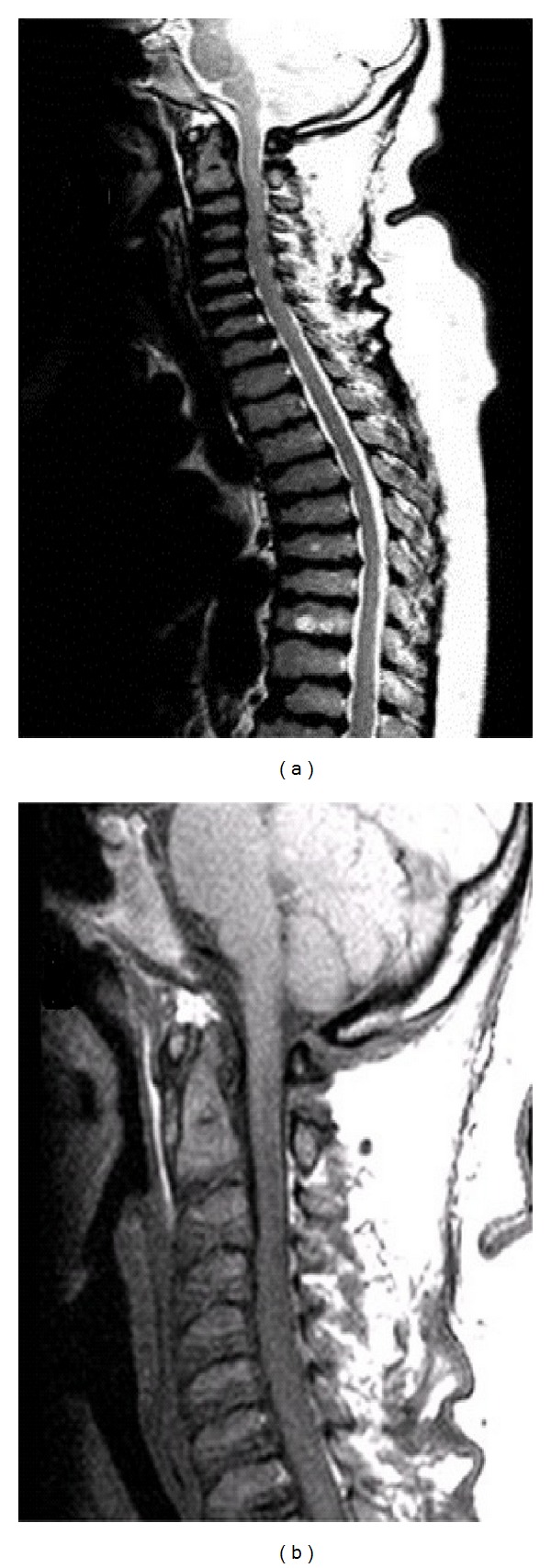
(a) MRI sagittal TSE T2 view of the cervico-thoracic spine. (b) MRI sagittal T1 view of the cervical spine showing hypoplasia of the odontoid process with a mild cord compression at the level of C2-C3.

**Figure 6:**
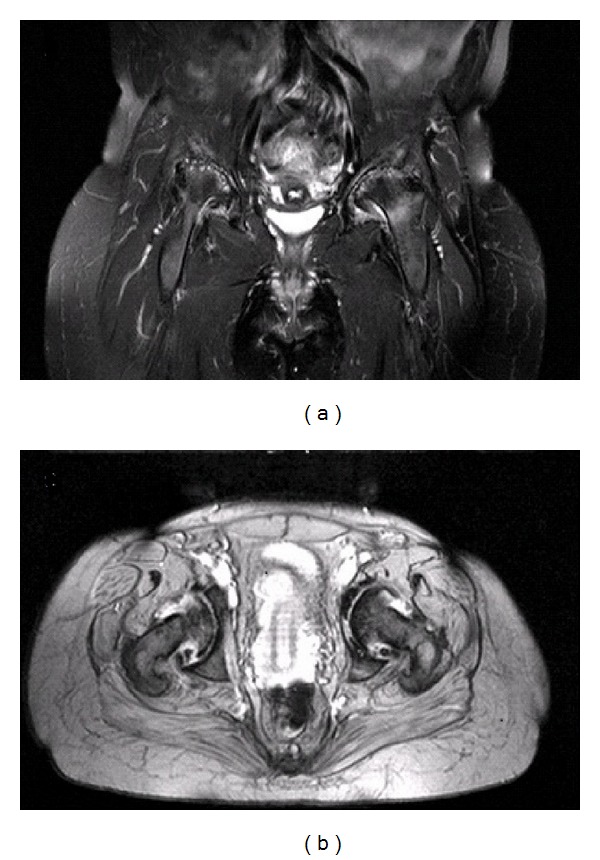
(a) MRI coronal FS view of the pelvis and (b) MRI axial ME 2D view of the pelvis.

**Figure 7:**
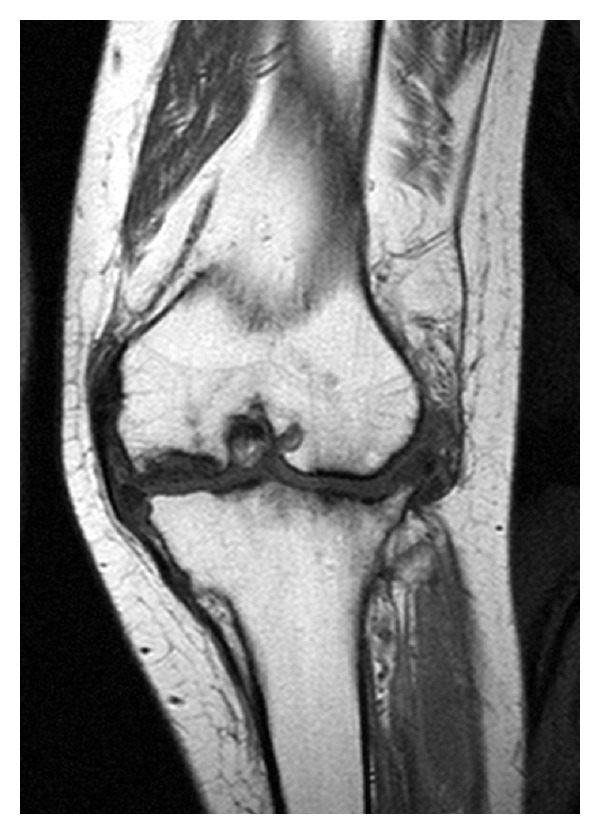
MRI T1 coronal view of the left knee.

**Figure 8:**
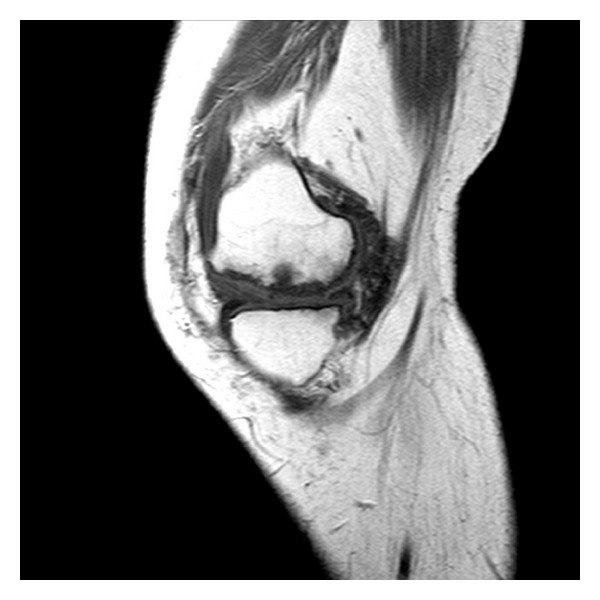
MRI T1 sagittal view of the left knee.

**Figure 9:**
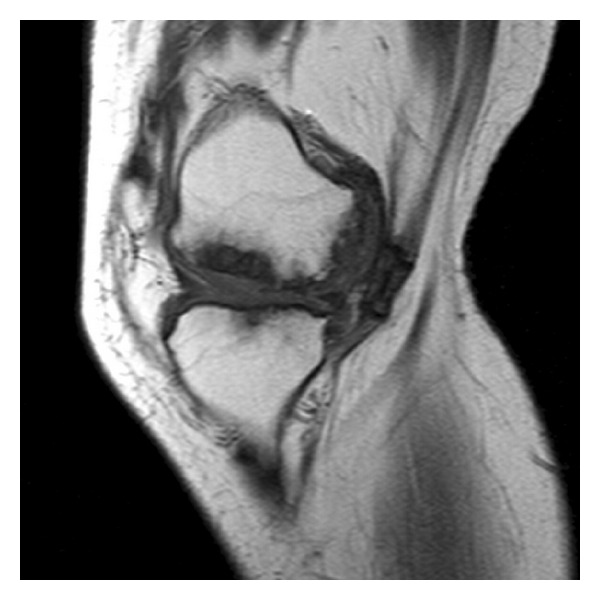
MRI T1 sagittal view of the right knee.

**Figure 10:**
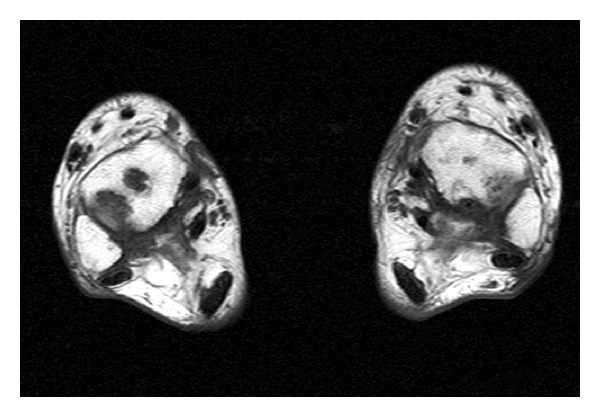
MRI TSE T1 axial view of the ankles at the level of the talar dome.

At the age of 40 years till now, the physical conditions of the patient have worsened for a further deterioration of her joints, with difficulty walking and recurrent pain. The neurological examination revealed hyporeflexia either at superior or at inferior limbs, paresthesias, and muscles fasciculations. With the aim to evaluate the locomotor system conditions of the patient, a full skeletal radiographic survey has been obtained (standing anterior posterior (AP) and lateral views of the entire spine, standing pelvis view, PA view of the chest, standing AP views of the knees). MR study with a 1,5 T superconducting system (Magneton Symphony, Siemens AG, Erlangen, Germany) of the entire column, hip, knees, and ankles was also performed, including coronal, sagittal and axial views. The radiographic and MRI assessment refers to a patient who did not undergo any other surgical procedures except for those carried out at 11 and 29 years. The AP radiographic view of the spine showed a mild thoraco-lumbar right scoliosis; the lateral view demonstrated a slight thoraco-lumbar kyphosis with irregular, flat and antero-posteriorly enlarged vertebral bodies particularly in mid-thoracic region, thoraco-lumbar junction and distal lumbar spine, produced by partial ossification of cartilaginous vertebral body (Figures 1(a), 1(b), and 1(c)). The cervical tract showed wedge shape of the vertebral bodies and an hypoplasia of the odontoid process (Figure 2). Roentgenographic findings of the chest included a relatively small size of her chest with oar-shaped ribs, (widening ribs anteriorly and narrowing posteriorly). The iliac wings of the pelvis were flared, with short femoral necks, flattered femoral epiphysis, and marked degenerative changes of the hip joints (Figure 3). In the lower extremity, the lower ends of the femur and the upper ends of the tibia were large with an evident genu valgus deformity. Severe degenerative changes of the knee joints were present with the signs of the previous left tibial osteotomy (Figure 4). MRI due to its multiplanar features confirmed radiographics findings adding further information on the degenerative alterations of the joints. Sagittal view of cervical and thoracic spine showed either the morphological alterations of the vertebral bodies or the hypoplasia of the odontoid process with a mild narrowing of the spinal canal and cord compression at the level of C2-C3 (Figures 5(a) and 5(b)). On dorsal tract platyspondyly, end plate irregularity, and anterior beaking of the vertebral bodies characteristics of dysostosis multiplex, were present. MRI of the pelvis in coronal view showed multiple abnormalities including oblique acetabular roof with, flattening of the femoral heads, and severe degenerative changes in the hip joints confirmed by axial views (Figures 6(a) and 6(b)). In addition to a genu valgus deformity, widening of the metaphysis and epiphyses, severe bilateral involvement of knees articular cartilage with multiple osteoarthritic changes were also detected. (Figures 7, 8, and 9) Similar findings were also carried out on ankle joints, in particular at the level of tibial epiphysis and talar dome (Figure 10).

## 3. Discussion

Morquio syndrome is a member of a group of inherited metabolic disorders collectively termed mucopolysaccharidoses (MPSs). The MPSs are caused by a deficiency of lysosomal enzymes required for the degradation of mucopolysaccharides or glycosaminoglycans (GAGs). Currently, eleven distinct single lysosomal enzyme deficiencies are known to cause seven recognized phenotypes of MPS [9]. All the MPSs are inherited in an autosomal recessive fashion, except Hunter syndrome (MPS type II) which is X linked [10]. 

The MPSs share a chronic progressive course with multisystem involvement, several physical features, laboratory findings, and radiographic abnormalities [5].

Patients with Morquio syndrome usually can be clinically distinguished from patients with other MPSs because they do not have coarse facial features or mental retardation and they have additional skeletal manifestations derived from a unique spondyloepiphyseal dysplasia and ligamentous laxity [4, 11]. These skeletal manifestations include odontoid hypoplasia, a striking short trunk dwarfism, and genu valgus. The patients with Morquio syndrome tend to have greater spine involvement with scoliosis, kiphosis, and severe gibbus, as well as platyspondyly, rib flaring, pectus carinatum, and ligamentous laxity [12, 13]. Odontoid hypoplasia is the most critical skeletal feature to be recognized in any patient with Morquio syndrome. In fact, both mortality and morbidity are related primarily to atlantoaxial subluxation resulting from the instability of the odontoid process [12].

The internationally estimated incidence of Morquio syndrome type IV covers a wide range: 1 case/75.000 in Northern Ireland, 1 case/200.000 in British Columbia, and 1 case/263.157 in Germany [14]. The exact incidence in Italy is unknown at present even if, according to the Italian Association of Mucopolysaccharidosis (AIMPS-Onlus), the estimated incidence is about 1 case/1250000 live births (unpublished data). Development of newborn screening strategies is underway. A very limited number of studies have been performed through the years regarding the skeletal status of patients with Morquio syndrome type IV B and only in very young people [5, 14]. The underlying defect in the mucopolysaccharidosis (MPSs) is inability to degrade glycosaminoglycans (GAGs). Dermatan sulfate, heparan sulfate, keratan sulfate (KS), and chondroitin sulfate are recognized as the main GAGs present in the tissues. In type IVB Morquio's syndrome the degradation of KS is defective because of beta-galactosidase (GLB1 gene) deficiency. KS is predominantly found in the cartilage and cornea, the major organs affected in Morquio syndrome [9]. Heparan and dermatan sulfate have a more generalized tissue distribution. Their normal metabolism in patients with Morquio syndrome spares these patients from mental retardation and disease manifestations observed in other types of MPSs. The specific mechanism by which excess storage of KS results in the skeletal dysplasia unique to Morquio syndrome remains unknown. The biology of KS is currently under investigation. Numerous KS-containing proteins have been identified and the elucidation of their functional roles will provide a better understanding of the pathophysiology of Morquio syndrome [9]. 

Although the diagnosis of Morquio syndrome is based on physical findings, blood enzymes, and a skin biopsy, radiographs and MR imaging provide useful information about the gravity of characteristic of skeletal and joint changes. Odontoid hypoplasia is the most critical feature to recognize and for this reason MRI of the neck must be used to determine if the upper vertebrae are underdeveloped, providing more accurate diagnosis on possible neurological risk conditions; moreover, it is helpful in confirming a possible cervical myelopathy and cord compression.

MRI study of lower limb joints did not demonstrate in our case the presence of osteonecrosis of the proximal and distal femur as sometimes described in patients with Morquio's disease. Actually, the general conditions of our patient are quite satisfactory, despite her joint pain and difficulty walking. A recent sight checkup revealed a slight accumulation of MPS in the anterior chamber of both eyes; she also complained of dental problems, especially on the incisors due to a laxity of alveolar ligaments. Although the moderate gravity of the skeletal alterations showed in our rare case, it does not appear to be classified as spondyloepiphyseal dysplasia (SED of Maroteaux) because of the presence of keratan sulfate [15]. The preservation of functionality is an increasing challenge in the treatment of patients with Morquio syndrome and maintenance of occupational performance should be defined as one of the main goals to be reached by the therapies used [16]. In conclusion, we would like to stress the uniqueness of our imaging collection; we believe that for the study of chronic progressive course with multijoint involvement of the Morquio disease, subsequent MRI imaging assessments can provide useful information, giving the patients a substantial impact about the evolution of this pathologic condition. It moreover allows the rheumatologist to play a major role ensuring control of the acute and chronic pain symptoms and the physiatrist to monitor bone and joint function, adopting the more appropriate rehabilitation strategies to be followed.
